# Exposure to BA.4/5 S protein drives neutralization of Omicron BA.1, BA.2, BA.2.12.1, and BA.4/5 in vaccine-experienced humans and mice

**DOI:** 10.1126/sciimmunol.ade9888

**Published:** 2022-11-15

**Authors:** Alexander Muik, Bonny Gaby Lui, Maren Bacher, Ann-Kathrin Wallisch, Aras Toker, Carla Iris Cadima Couto, Alptekin Güler, Veena Mampilli, Geneva J. Schmitt, Jonathan Mottl, Thomas Ziegenhals, Stephanie Fesser, Jonas Reinholz, Florian Wernig, Karla-Gerlinde Schraut, Hossam Hefesha, Hui Cai, Qi Yang, Kerstin C. Walzer, Jessica Grosser, Stefan Strauss, Andrew Finlayson, Kimberly Krüger, Orkun Ozhelvaci, Katharina Grikscheit, Niko Kohmer, Sandra Ciesek, Kena A. Swanson, Annette B. Vogel, Özlem Türeci, Ugur Sahin

**Affiliations:** ^1^ BioNTech, An der Goldgrube 12, 55131 Mainz, Germany.; ^2^ Pfizer, 401 N. Middletown Rd., Pearl River, NY 10960, USA.; ^3^ Institute for Medical Virology, University Hospital, Goethe University Frankfurt, 60596 Frankfurt am Main, Germany.; ^4^ DZIF – German Centre for Infection Research, External Partner Site, 60596 Frankfurt am Main, Germany.; ^5^ HI-TRON – **Helmholtz Institute for Translational Oncology Mainz** by DKFZ, Obere Zahlbacherstr. 63, 55131 Mainz, Germany.; ^6^ TRON gGmbH – Translational Oncology at the University Medical Center of the Johannes Gutenberg University, Freiligrathstraße 12, 55131 Mainz, Germany.

## Abstract

The SARS-CoV-2 Omicron variant and its sublineages show pronounced viral escape from neutralizing antibodies elicited by vaccination or prior SARS-CoV-2 variant infection owing to over 30 amino acid alterations within the spike (S) glycoprotein. Breakthrough infection of vaccinated individuals with Omicron sublineages BA.1 and BA.2 is associated with distinct patterns of cross-neutralizing activity against SARS-CoV-2 variants of concern (VOCs). In continuation of our previous work, we characterized the effect of Omicron BA.4/BA.5 S glycoprotein exposure on the neutralizing antibody response upon breakthrough infection in vaccinated individuals and upon variant-adapted booster vaccination in mice. We found that immune sera from triple mRNA-vaccinated individuals with subsequent breakthrough infection during the Omicron BA.4/BA.5 wave showed cross-neutralizing activity against previous Omicron variants BA.1, BA.2, BA.2.12.1, and BA.4/BA.5 itself. Administration of a prototypic BA.4/BA.5-adapted mRNA booster vaccine to mice following SARS-CoV-2 wild-type strain-based primary immunization is associated with broader cross-neutralizing activity than a BA.1-adapted booster. While the Omicron BA-1-adapted mRNA vaccine in a bivalent format (wild-type + BA.1) broadens cross-neutralizing activity relative to the BA.1 monovalent booster, cross-neutralization of BA.2 and descendants is more effective in mice boosted with a bivalent wild-type + BA.4/BA.5 vaccine. In naïve mice primary immunization with the bivalent wild-type + Omicron BA.4/BA.5 vaccine induces strong cross-neutralizing activity against Omicron VOCs and previous variants. These findings suggest that when administered as boosters, mono- and bivalent Omicron BA.4/BA.5-adapted vaccines enhance neutralization breadth, and that the bivalent version also has the potential to confer protection to individuals with no pre-existing immunity against SARS-CoV-2.

## Teaser

Immune sera from BA.4/BA.5 breakthrough infected individuals and BA.4/5 booster-vaccinated mice broadly neutralize Omicron sublineages.

## INTRODUCTION

SARS-CoV-2 Omicron and its sublineages have had a major impact on the epidemiological landscape of the COVID-19 pandemic since initial emergence in November 2021 (*1*, *2*). Significant alterations in the spike (S) glycoprotein of the first Omicron variant BA.1 leading to the loss of many neutralizing antibody epitopes (*3*) rendered BA.1 capable of partially escaping previously established SARS-CoV-2 wild-type strain (Wuhan-Hu-1)-based immunity (*4*, *5*). Hence, breakthrough infection of vaccinated individuals with Omicron are more common than with previous VOCs. While Omicron BA.1 was displaced by the BA.2 variant in many countries around the globe, other variants such as BA.1.1 and BA.3 temporarily and/or locally gained momentum but did not become globally dominant (*6*, *7*). Omicron BA.2.12.1 subsequently displaced BA.2 to become dominant in the United States, whereas BA.4 and BA.5 displaced BA.2 in Europe, parts of Africa, and Asia/Pacific (*7*–*9*). Currently, Omicron BA.5 is dominant globally, including in the United States (*10*).

Omicron has acquired numerous alterations (amino acid exchanges, insertions, or deletions) in the S glycoprotein, among which some are shared between all Omicron VOCs while others are specific to one or more Omicron sublineages (fig S1). Antigenically, BA.2.12.1 exhibits high similarity with BA.2 but not BA.1, whereas BA.4 and BA.5 differ considerably from their ancestor BA.2 and even more so from BA.1, in line with their genealogy (*11*). Major differences of BA.1 from the remaining Omicron VOCs include Δ143–145, L212I, or ins214EPE in the S glycoprotein N-terminal domain and G446S or G496S in the receptor binding domain (RBD). Amino acid changes T376A, D405N, and R408S in the RBD are in turn common to BA.2 and its descendants but not found in BA.1. In addition, some alterations are specific for individual BA.2-descendant VOCs, including L452Q for BA.2.12.1 or L452R and F486V for BA.4 and BA.5 (BA.4 and BA.5 encode for the same S sequence). Most of these shared and VOC-specific alterations were shown to play an important role in immune escape from monoclonal antibodies and polyclonal sera raised against the wild-type S glycoprotein. In particular, the BA.4/BA.5-specific alterations are strongly implicated in immune escape of these VOCs (*12*, *13*).

We and others have previously reported that Omicron BA.1 and BA.2 breakthrough infection of individuals previously immunized with wild-type strain-based vaccines was associated with potent neutralizing activity against Omicron BA.1, BA.2 and previous VOCs (*14*–*17*). A considerable boost of Omicron BA.2.12.1 and BA.4/BA.5 neutralization was evident in BA.2 but not BA.1 breakthrough cases, albeit with titers against BA.4/BA.5 remaining lower than titers against previous Omicron VOCs (*17*, *18*). Analyses of immune sera from clinical trial participants showed that sera from SARS-CoV-2 naïve individuals vaccinated with an Omicron BA.1-adapted mRNA vaccine as a fourth dose showed superior neutralizing responses against BA.1 compared to sera from individuals boosted with the respective wild-type strain-based mRNA vaccine BNT162b2 (*19*) or mRNA-1273 (*20*). However, the BA.1-adapted booster was not associated with increased BA.4/BA.5 cross-neutralization when compared to wild-type-boosted individuals (*20*).

Given the current global predominance of the highly contagious Omicron BA.5, we investigated whether exposure to Omicron BA.4/BA.5 S glycoprotein would mediate a cross-neutralizing antibody response against relevant Omicron VOCs. We assessed the breadth of neutralizing activity against Omicron VOCs in immune sera from vaccinated individuals with Omicron BA.4/BA.5 breakthrough infection and in sera from mice that received Omicron BA.4/BA.5-adapted booster vaccines following primary immunization with BNT162b2. We also evaluated the breadth of neutralizing activity following immunization of SARS-CoV-2 naïve mice with Omicron BA.4/BA.5-adapted vaccines. We found that exposure to Omicron BA.4/BA.5 S glycoprotein mediates robust neutralization of all currently or previously predominant Omicron VOCs in humans and mice. We believe that our data may contribute to the understanding of the effect of exposure to different variant S glycoproteins on sera cross-neutralization activity and to the design of vaccination strategies.

## RESULTS

### Omicron BA.4/BA.5 breakthrough infection of triple mRNA-vaccinated individuals results in Omicron sublineage cross-neutralizing activity

**Fig. 1.**
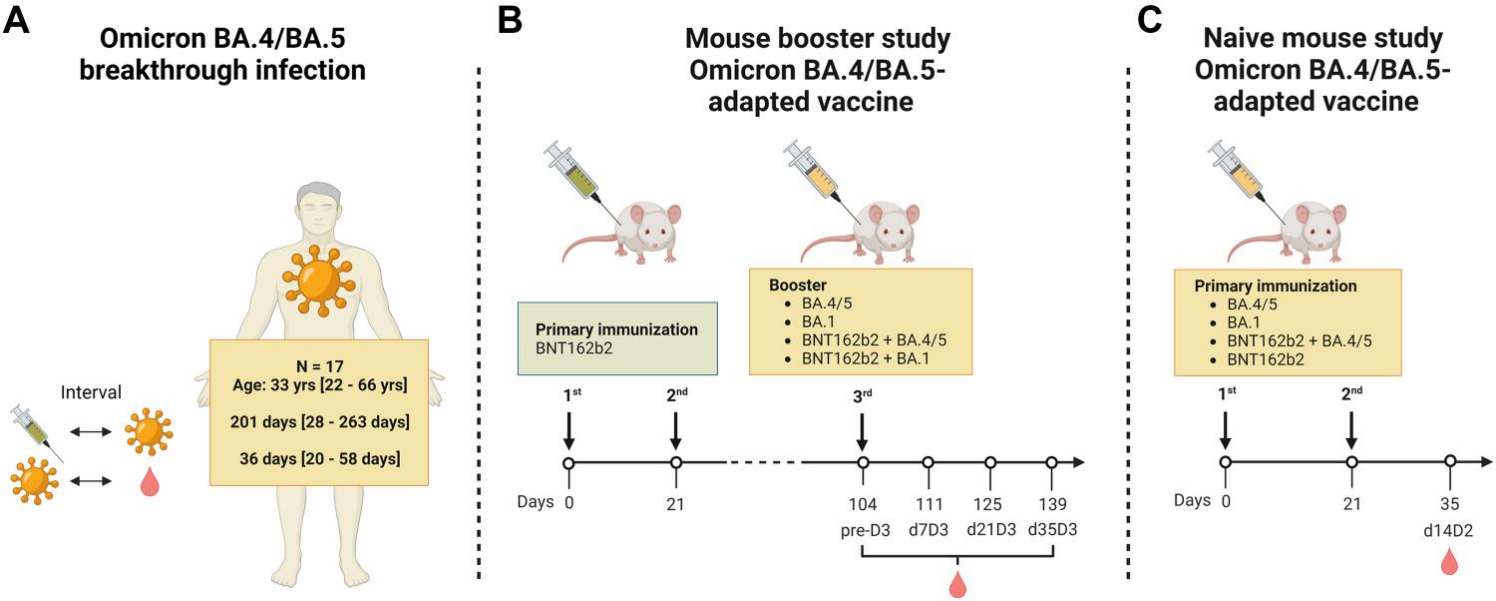
Study design. (**a**)The effect of Omicron BA.4/BA.5 breakthrough infection on the serum neutralizing activity was evaluated in individuals vaccinated with three doses of mRNA COVID-19 vaccine (BNT162b2/mRNA-1273 homologous or heterologous regimens) who subsequently experienced an infection with Omicron BA.4 or BA.5. The intervals between vaccination, breakthrough infection and sampling are indicated as median/range. (**b**) Effects of prototypic Omicron BA.4/BA.5-adapted booster vaccines on serum neutralizing activity were investigated in mice vaccinated twice with BNT162b2, followed by a booster dose of BA.4/BA.5-adapted or BA.1 adapted vaccines. Adapted vaccines were administered either as monovalent boosters or bivalent boosters with BNT162b2. Neutralizing activity was assessed before (pre-D3) and 7, 21, and 35 days after the booster (d7D3, d21D3, d35D3, respectively). (**c**) Effects of prototypic Omicron BA.4/BA.5-adapted vaccines on serum neutralizing activity were investigated in naïve mice vaccinated twice with BA.4/BA.5-adapted (monovalent or bivalent) or BA.1-adapted (monovalent) vaccines, or BNT162b2. Neutralizing activity was assessed 14 days after administration of the second dose (d14D2). **Comparably high RNA purity and integrity, and expression of antigens *in vitro* were confirmed for BNT162b2 and Omicron-adapted vaccines** (fig S4a-b).

We investigated the effect of Omicron BA.4/BA.5 breakthrough infection of individuals vaccinated with three prior doses of mRNA COVID-19 vaccine (BNT162b2/mRNA-1273 homologous or heterologous regimens) on serum neutralizing activity against SARS-CoV-2 variants (mRNA-Vax^3^ + BA.4/BA.5, n = 17, Fig. 1a and S2, Tables S1 and S2). Three cohorts were included for reference: triple mRNA vaccinated individuals with an Omicron BA.2 breakthrough infection (mRNA-Vax^3^ + BA.2, n = 19) or with an Omicron BA.1 breakthrough infection (mRNA-Vax^3^ + BA.1, n = 14), and BNT162b2 triple-vaccinated individuals who were SARS-CoV-2-naïve at the time of sampling (BNT162b2^3^, n = 18, fig. S2 and Table S1). Data for the reference cohorts were previously published and are used here for benchmarking (*14*, *17*).

Serum neutralizing activity was tested in a well-characterized pseudovirus neutralization test (pVNT) (*17*, *21*, *22*) by determining 50% pseudovirus neutralization (pVN_50_) geometric mean titers (GMTs) with pseudoviruses bearing the S glycoproteins of the SARS-CoV-2 wild-type strain or Omicron BA.1, BA.2, BA.2.12.1, and BA.4/BA.5 (BA.4 and BA.5 are identical in their S glycoprotein sequence). In addition, we assayed SARS-CoV (herein referred to as SARS-CoV-1) for potential pan-sarbecovirus neutralizing activity (*23*). As an orthogonal test system, we used a live SARS-CoV-2 neutralization test (VNT) that analyzes neutralization during multicycle replication of authentic virus (SARS-CoV-2 wild-type strain and Omicron BA.1, BA.2, and BA.4) with immune serum present during the entire test period.

**Fig. 2.**
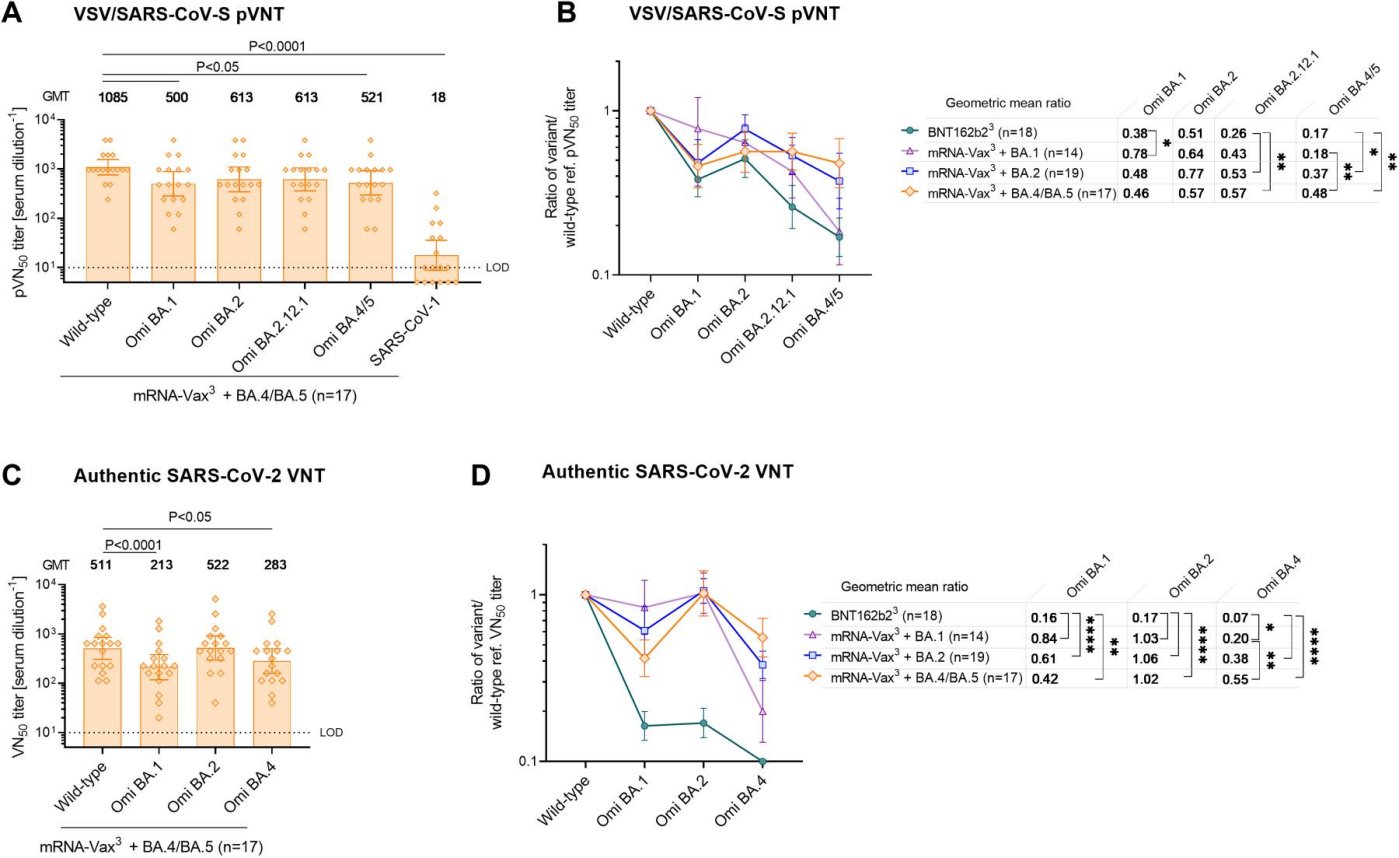
Omicron BA.4/BA.5 breakthrough infection of triple mRNA vaccinated individuals mediates cross-neutralization of Omicron sublineages. Cohorts and serum sampling as described in Fig. S2. (**a**) 50% pseudovirus neutralization (pVN_50_) geometric mean titers (GMTs) in sera of mRNA-Vax^3^ + BA.4/BA.5 against the indicated SARS-CoV-2 variants of concern (VOCs) or SARS-CoV-1 pseudoviruses. Values above bars represent group GMTs. The non-parametric Friedman test with Dunn’s multiple comparisons correction was used to compare the wild-type strain neutralizing group GMTs with titers against the indicated variants and SARS-CoV-1. Multiplicity-adjusted p values are shown. (**b**) SARS-CoV-2 VOC pVN_50_ GMTs normalized against the wild-type strain (ratio VOC to wild-type) of mRNA-Vax^3^ + BA.4/BA.5 and the reference cohorts. Group geometric mean ratios with 95% confidence intervals are shown. The non-parametric Kruskal-Wallis test with Dunn’s multiple comparisons correction was used to compare the VOC GMT ratios between cohorts. ****, P < 0.0001; **, P < 0.01; *, P < 0.05. (**c**) 50% virus neutralization (VN_50_) GMTs in sera of mRNA-Vax^3^ + BA.4/BA.5 against the indicated SARS-CoV-2 VOCs. Statistical analysis was conducted as in (a). (**d**) SARS-CoV-2 VOC VN_50_ GMTs normalized against the wild-type strain VN_50_ GMT (ratio VOC to wild-type). Statistical analysis was conducted as in (b). For titer values below the limit of detection (LOD), LOD/2 values were plotted.

In the pVNT, sera from the Omicron BA.4/BA.5 breakthrough infection cohort (mRNA-Vax^3^ + BA.4/BA.5) robustly neutralized the wild-type strain and all tested Omicron VOCs (Fig. 2a). The pVN_50_ GMTs against Omicron BA.2 and BA.2.12.1 pseudoviruses were within a 2-fold range of the GMT against the wild-type strain (GMTs 613 against Omicron vs. GMT 1085 against wild-type). Neutralization of BA.1 and BA.4/5 (GMTs 500–521) was broadly similar to that of BA.2, and the reduction relative to the wild-type strain significant (p < 0.05) yet also within a ~ 2-fold range. The GMT against SARS-CoV-1 was significantly lower (p < 0.0001; >50-fold lower than wild-type).

To compare mRNA-Vax^3^ + BA.4/BA.5 to the reference cohorts with Omicron BA.1 or BA.2 breakthrough infection (mRNA-Vax^3^ + BA.1 and mRNA-Vax^3^ + BA.2) and triple BNT162b2-vaccinated SARS-CoV-2 naïve individuals (BNT162b2^3^), we normalized the VOC pVN_50_ GMTs against those of the wild-type strain to allow for assessment of neutralization breadth irrespective of the magnitude of antibody titers, which expectedly differs between triple-vaccinated individuals without versus with a breakthrough infection (*14*, *17*). While BNT162b2^3^ sera mediated considerable cross-neutralization of Omicron BA.1 and BA.2, breakthrough infection with Omicron BA.1 was associated with significantly (p < 0.05) higher neutralization of the homologous strain (Fig. 2b). A similar trend only short of statistical significance (p = 0.06) was observed for BA.2 neutralization after BA.2 breakthrough infection. Importantly, cross-neutralization of BA.4/5 was significantly (p < 0.01) stronger in the mRNA-Vax^3^ + BA.4/5 cohort (GMT ratio 0.48) compared to BNT162b2^3^ and to mRNA-Vax^3^ + BA.1 (GMT ratios 0.17 and 0.18, respectively). Cross-neutralization of BA.4/5 was also stronger than in the mRNA-Vax^3^ + BA.2 cohort; the difference was less pronounced (GMT ratios 0.48 versus 0.37, respectively) and not statistically significant. Cross-neutralization of Omicron BA.1 and BA.2 was maintained at comparatively higher levels in the mRNA-Vax^3^ + BA.4/BA.5 cohort (GMT ratios 0.46 and 0.57, respectively). In conclusion, BA.4/BA.5 breakthrough infection resulted in cross-neutralization of Omicron BA.1, BA.2, BA.2.12.1 and of BA.4/BA.5 itself (GMT ratios ≥0.46). Although BA.1 and BA.2 breakthrough infection also resulted in neutralization of BA.1, BA.2, and BA.2.12.1, there was less potent cross-neutralization against the subsequent BA.4/BA.5 variant.

The authentic live SARS-CoV-2 virus neutralization assay largely confirmed the pVNT assay findings shown in Fig. 2a-b. 50% virus neutralization (VN_50_) GMT in Omicron BA.4/BA.5 breakthrough sera against BA.2 was comparable to that against the wild-type strain (Fig. 2c). BA.4 and BA.1 neutralization were significantly reduced yet also within a 2 to 2.5-fold range. The GMTs normalized against the wild-type strain showed robust cross-neutralization of Omicron BA.1, BA.2, and BA.4 by mRNA-Vax^3^ + BA.4/BA.5 sera (GMT ratios 0.42, 1.02, and 0.55, respectively), whereas BA.4 cross-neutralization was considerably less efficient in mRNA-Vax^3^ + BA.1 (GMT ratio 0.20) and mRNA-Vax^3^ + BA.2 (GMT ratio 0.38) sera (Fig. 2d). In summary, our findings showed association of Omicron BA.4/BA.5 breakthrough infection with cross-neutralizing activity against BA.4/5 itself and previously dominant Omicron VOCs.

### Booster immunization with an Omicron BA.4/BA.5 S glycoprotein adapted mRNA vaccine drives Omicron sublineage cross-neutralization in BNT162b2 double-vaccinated mice

The heightened neutralization breadth seen after Omicron BA.4/BA.5 breakthrough infection suggested that variant-adapted vaccines based on the Omicron BA.4/5 S glycoprotein sequence may elicit a recall response with broader cross-neutralization than those based on Omicron BA.1. We tested this hypothesis by booster studies in BNT162b2-preimmunized mice (Fig. 1b). Mice were administered a primary 2-dose series of BNT162b2 on days 0 and 21 and a third dose of either BNT162b2, or variant-adapted prototypic vaccines (fig. S3) on day 104 (3.5 months after dose 2). The variant adapted vaccines were either monovalent encoding Omicron BA.1 or BA.4/5 S glycoprotein (1 μg), or bivalent combining either of these Omicron variant S glycoprotein sequences with BNT162b2 (0.5 μg each). Neutralizing titers against pseudoviruses expressing the wild-type strain, Omicron BA.1, BA.2, BA.2.12.1, or BA.4/5 S glycoprotein were determined in pVNT assays using sera drawn before the booster (day 104, pre-dose 3[D3]) and on days 7, 21, and 35 after the third dose (d7D3, d21D3 and d35D3). The live SARS-CoV-2 neutralization test was used as an orthogonal test system to confirm the observed pseudovirus neutralizing activity post-boost on d35D3.

In sera drawn on pre-D3, pVN_50_ GMTs of the groups dedicated for the various boosters against all tested variants were largely comparable (fig. S5a). pVN_50_ GMTs against the Omicron sublineage pseudoviruses were significantly lower than those against the wild-type pseudovirus. Titers against Omicron BA.1 and BA.2 were 3 to 11-fold lower than those against the wild-type strain (GMT ratios ≤0.32, fig. S5b). GMTs against BA.2.12.1 and BA.4/5 were 10 to 25-fold lower than those against wild-type (GMT ratios ≤0.10).

**Fig. 3.**
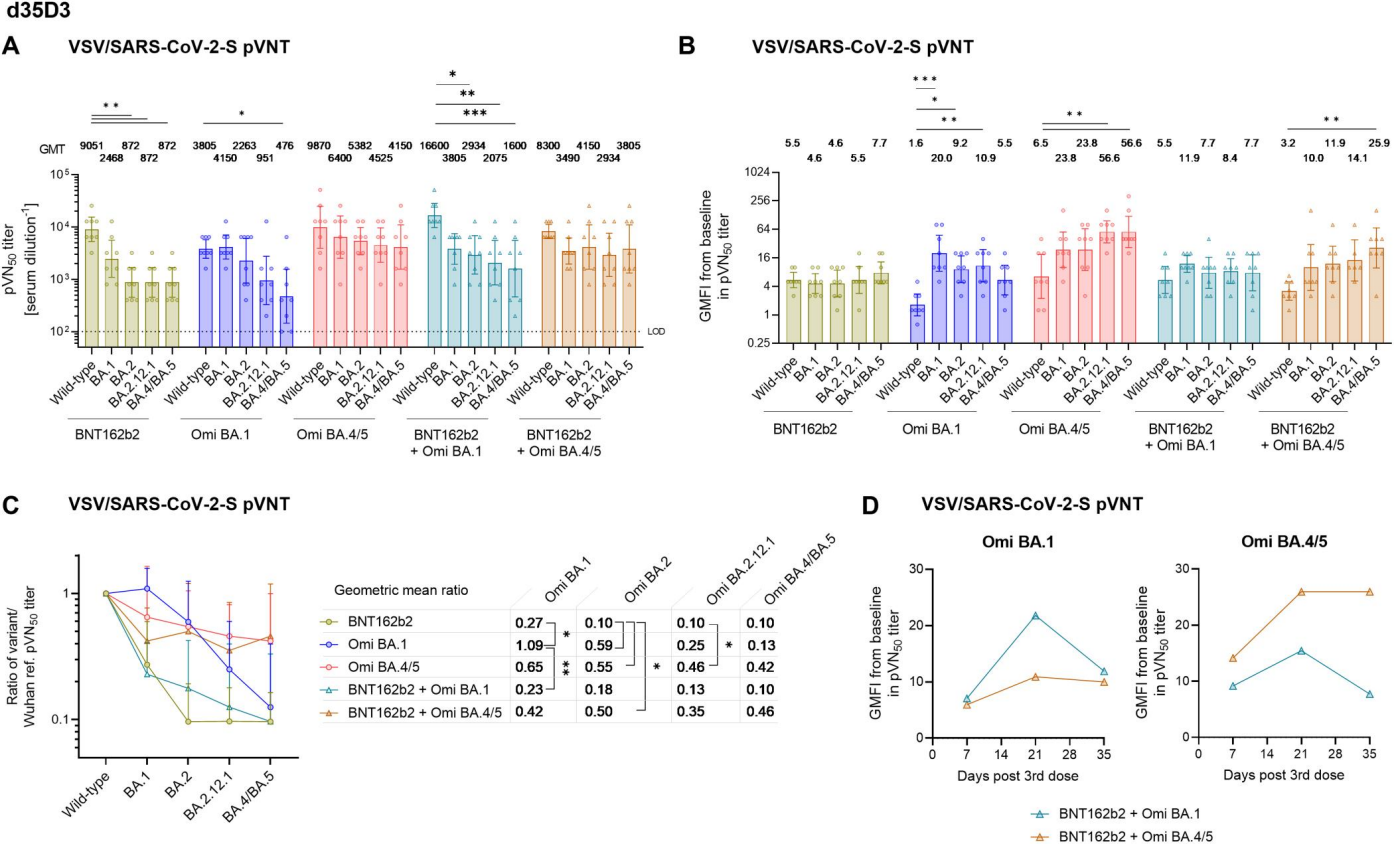
Booster immunization with an Omicron BA.4/BA.5 S glycoprotein adapted RNA- vaccine mediates broad Omicron neutralization in double-vaccinated mice. BALB/c mice (n = 8) were injected intramuscularly with two doses of 1 μg BNT162b2 21 days apart, and a third dose of either BNT162b2 (1 μg) or the indicated monovalent (1 μg) or bivalent (0.5 μg of each component) Omicron BA.1 or BA.4/5-adapted vaccines 104 days after the first vaccination. (**a**) 50% pseudovirus neutralization (pVN_50_) geometric mean titers (GMTs) against the indicated SARS-CoV-2 variants of concern (VOCs) in sera collected 35 days after the third vaccination (d35D3). Values above bars represent group GMTs. (**b**) Geometric mean fold-increase (GMFI) of pVN_50_ titers on d35D3 relative to baseline titers before the third vaccination. Values above bars represent group GMFIs. The non-parametric Friedman test with Dunn’s multiple comparisons correction was used to compare the wild-type strain neutralizing group GMTs and group GMFI with those against the indicated variants. Multiplicity-adjusted p values are shown. ***, P < 0.01; **, P < 0.01; *, P < 0.05. (**c**) SARS-CoV-2 VOC pVN_50_ GMTs normalized against the wild-type strain pVN_50_ GMT (ratio VOC to wild-type). Group geometric mean ratios are shown. The non-parametric Kruskal-Wallis test with Dunn’s multiple comparisons correction was used to compare the VOC GMT ratios between cohorts. Significance levels are summarized as above. (**d**) GMFI of pVN_50_ titers against BA.1 and BA.4/5 over time relative to baseline titers before the booster vaccination with BNT162b2/BA.1 or BNT162b2/BA.4/5 bivalent vaccines. For titer values below the limit of detection (LOD), LOD/2 values were plotted. Error bars represent 95% confidence intervals.

On d7D3 and d21D3, neutralizing titers had increased substantially across groups and against all tested variants (fig. S6 and S7). Titers remained high on d35D3, the last time point analyzed in this study (Fig. 3). In sera from BNT162b2 boosted mice, strong neutralizing activity against the wild-type strain was observed, whereas pVN_50_ GMTs against Omicron variants were substantially lower (Fig. 3a). The Omicron BA.1 booster elicited considerable neutralization titers against BA.1 and the wild-type strain, while the pVN_50_ GMTs against Omicron BA.2.12.1 and BA.4/5 were considerably lower. In particular, the GMT against BA.4/5 were significantly reduced (8-fold compared to those against wild-type). In contrast, administration of the BA.4/5 booster elicited broad cross-neutralizing activity against all Omicron variants and the wild-type strain. Sera from mice that received the BNT162b2/BA.1 bivalent booster had a high pVN_50_ GMT against the wild-type strain, whereas the GMTs against Omicron sublineages were overall reduced with titers against BA.2, BA.2.12.1 and BA.4/5 being significantly lower than those against the wild-type strain. The BNT162b2/Omicron BA.4/5 bivalent booster gave rise to high titers against the wild-type strain, comparable to the BNT162b2 monovalent booster. Omicron neutralization was broadly comparable across all VOCs, with pVN_50_ GMTs modestly below that against the wild-type strain.

We evaluated the fold-changes in pVN_50_ GMTs detected on d35D3 relative to the baseline GMTs determined before administration of the third dose. BNT162b2 comparably increased neutralization of all tested variants (pVN_50_ GMTs 5 to 8-fold higher than at pre-D3). The monovalent BA.1 booster significantly enhanced titers against Omicron BA.1, BA.2 and BA.2.12.1 relative to those against wild-type, with the most pronounced effect detected for the neutralization of the homologous VOC (20-fold increase) (Fig. 3b). The monovalent BA.4/5 booster strongly potentiated neutralizing activity against BA.2.12.1 and BA.4/5 (57-fold, p < 0.01 compared to wild-type) and also substantially boosted BA.1 and BA.2 neutralization (increases of 24-fold). The BNT162b2/BA.1 bivalent vaccine showed an overall similar response pattern as the BA.1 monovalent vaccine albeit with stronger potentiation of titers against the wild-type strain (6-fold vs. 2-fold) and with further enhanced titers against Omicron BA.1 (12-fold increase). The BNT162b2/BA.4/5 bivalent vaccine triggered significant potentiation of titers against Omicron BA.4/5 (26-fold) compared to those against wild-type, and also considerably boosted BA.1, BA.2, and BA.2.12.1 neutralization (10 to 14-fold). Potentiation of titers against individual Omicron sublineages by the BA.4/5 bivalent vaccine followed a similar pattern as with the BA.4/5 monovalent vaccine, albeit with modestly attenuated fold-increases in titers.

To compare neutralization breadth across groups irrespective of the magnitude of antibody titers, we normalized the VOC pVN_50_ GMTs against the wild-type strain and found that the Omicron BA.4/5 booster vaccine mediated cross-neutralization of all tested Omicron sublineages (GMT ratios ≥0.42) (Fig. 3c). The GMT ratios for Omicron BA.2 and BA.2.12.1 were significantly higher than those in the BNT162b2 boosted group. In contrast, the BA.1 booster vaccine robustly neutralized BA.1 (GMT ratio 1.09), whereas GMT ratios for BA.2.12.1 (0.23) and BA.4/5 (0.13) were substantially lower. The bivalent BNT162b2/BA.1 vaccine mediated less efficient neutralization of BA.1 (GMT ratio 0.23) compared to the BA.1 monovalent booster, and cross-neutralization of BA.4/5 was comparable (GMT ratios 0.13 and 0.10). The bivalent BNT162b2/BA.4/5 vaccine mediated stronger cross-neutralization of Omicron BA.2, BA.2.12.1, and BA.4/5 compared to BNT162b2/BA.1, with GMT ratios (between 0.35 and 0.50) comparable to those of the BA.4/5 monovalent vaccine.

The Omicron BA.4/5 monovalent and BNT162b2/BA.4/5 bivalent boosters mediated a similarly broad neutralizing activity on d7D3 (fig. S6a). Neutralizing activity against Omicron sublineages was significantly lower than against the wild-type strain in the groups boosted with the monovalent BA.1 and the bivalent BNT162b2/BA.1 vaccine. Accordingly, potentiation of pre-boost titers at d7D3 was similarly strong or stronger in mice boosted with BA.4/5 compared to BA.1 vaccines, both in the monovalent and the bivalent format (fig. S6b). Neutralization breadth was comparable in sera of mice boosted with BA.4/5 vaccines, whereas sera from BA.1 boosted mice exhibited considerably lower cross-neutralization of BA.2, BA.2.12.1 and BA.4/5 (fig S6c). A similar trend was observed on d21D3 for group GMTs and fold-increases (fig. S7a-b). Cross-neutralization of Omicron sublineages by the BA.4/5 bivalent booster was less efficient than with the monovalent BA.4/5 booster, and only slightly stronger than that of the BA.1 bivalent booster at this time point (fig. S7c).

The authentic live SARS-CoV-2 virus neutralization assay largely confirmed the pVNT assay findings. Sera from groups boosted with monovalent or bivalent BA.4/5 vaccines exhibited broader cross-neutralizing activity than those receiving the respective BA.1 vaccines and BNT162b2 (fig. S8). In the VNT assay, cross-neutralization of Omicron sublineages mediated by the bivalent BNT162b2/BA.4/5 booster was less pronounced compared to the monovalent BA.4/5 booster.

Taken together, the BA.4/5 monovalent booster was superior to the BA.1 monovalent booster in mediating broader cross-neutralizing activity. While the bivalent BNT162b2/BA.1 booster broadened cross-neutralizing activity relative to the BA.1 monovalent booster, cross-neutralization of Omicron sublineages was overall more effective in mice boosted with the BNT162b2/BA.4/5 bivalent vaccine. While potentiation of pre-boost BA.1 titers was stronger in mice boosted with the BNT162b2/BA.1 bivalent vaccine with peak titers on d21D3, similar fold-increases relative to pre-boost were detected on d7D3 and d35D3 in groups with the bivalent BNT162b2/BA.1 and BNT162b2/BA.4/5 boosters (Fig. 3d). In contrast, the bivalent BNT162b2/BA.4/5 booster was associated with a stronger potentiation of titers against BA.4/5 compared to the BNT162b2/BA.1 bivalent booster at all time points analyzed, which remained constant between d21D3 and d35D3. In conclusion our findings suggest that a booster with an Omicron BA.4/5 S glycoprotein adapted monovalent or bivalent vaccine following primary immunization with a wild-type strain-based vaccine may elicit superior neutralizing activity against current and previously dominant Omicron VOCs as compared to a BA.1 S glycoprotein-based booster.

### Immunization with an Omicron BA.4/BA.5 S glycoprotein adapted mRNA vaccine drives cross-neutralization of Omicron sublineages in previously unvaccinated mice

**Fig. 4.**
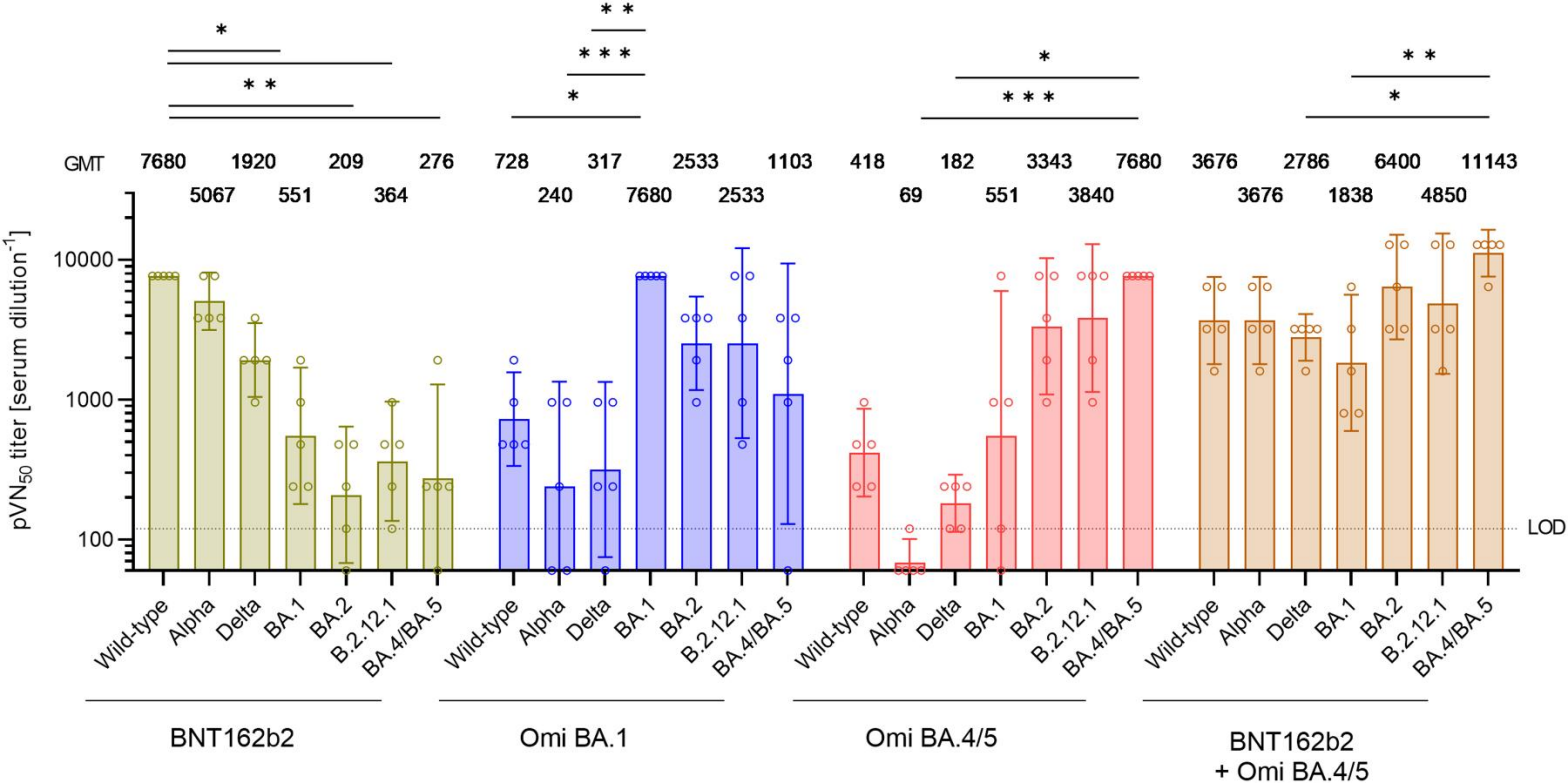
Immunization with an Omicron BA.4/BA.5 S glycoprotein supplemented BNT162b2 mRNA vaccine drives broad SARS-CoV-2 VOC cross-neutralization in previously unvaccinated mice. Naïve BALB/c mice (n = 5) were injected intramuscularly with two doses of either BNT162b2 (1 μg) or the indicated monovalent (1 μg) or bivalent (0.5 μg of each component) Omicron BA.1 or BA.4/5-adapted vaccines, 21 days apart. 50% pseudovirus neutralization (pVN_50_) geometric mean titers (GMTs) against the indicated SARS-CoV-2 variants of concern (VOCs) in sera collected 14 days after the second vaccination (d14D2). Values above bars represent group GMTs. Error bars represent 95% confidence interval. The non-parametric Friedman test with Dunn’s multiple comparisons correction was used to compare the vaccine strain neutralizing group GMTs (Omicron BA.4/5 in case of the bivalent vaccine) with titers against the wild-type strain and/or remaining variants. Multiplicity-adjusted p values are shown. ***, P < 0.01; **, P < 0.01; *, P < 0.05. For titer values below the limit of detection (LOD), LOD/2 values were plotted.

We sought to understand the impact of immunization with the Omicron-adapted vaccine on the serum neutralizing capacity of mice with no pre-existing immune response against SARS-CoV-2. Naïve mice were immunized twice, at day 0 and day 21 with either BNT162b2, with BA.1 or BA.4/5 S glycoprotein adapted monovalent vaccines, or the bivalent BNT162b2/BA.4/5 vaccine (Fig. 1c). Neutralizing titers against pseudoviruses expressing the wild-type strain S glycoprotein, Alpha or Delta, or Omicron BA.1, BA.2, BA.2.12.1, or BA.4/5 were determined in pVNT assays using sera drawn 14 days after the second immunization (d14D2). In sera from BNT162b2 immunized mice, robust neutralizing activity against the wild-type strain, Alpha, and Delta was observed, whereas pVN_50_ GMTs against Omicron variants were significantly (14 to 37-fold, p < 0.05) lower than those against wild-type (Fig. 4). Immunization with the Omicron BA.1 monovalent vaccine led to strong neutralization of BA.1. In these sera, robust neutralization of Omicron BA.2 and BA.2.12.1 was also detected, while the pVN_50_ GMTs against the wild-type strain and remaining VOCs were considerably (7 to 32-fold) lower than BA.1. Immunization with the Omicron BA.4/5 monovalent vaccine led to high neutralization of BA.4/5. In these sera, robust neutralization of Omicron BA.2 and BA.2.12.1 was also detected, while the pVN_50_ GMTs against the wild-type strain and previous VOCs were considerably (14 to >42-fold) lower than BA.4/5. Immunization with the BNT162b2/BA.4/5 bivalent vaccine resulted in high neutralizing activity not only against BA.4/5, but also against the wild-type strain and Alpha, Omicron BA.2 and BA.2.12.1 VOCs (within a 3-fold range of BA.4/5). While titers against Delta and Omicron BA.1 were significantly lower than those against BA.4/5, neutralization breadth in sera of BNT162b2/BA.4/5 vaccinated mice was superior to the remaining groups (titers against all variants within a 6-fold range, compared to reductions of up to >30-fold in all other groups).

These results show that a monovalent vaccine in naïve animals induces a high neutralizing antibody response mostly in a variant-specific manner, whereas a bivalent vaccine provided enhanced neutralization breadth across the wild-type strain and VOCs.

## DISCUSSION

Here, we report that BA.4/BA.5 breakthrough infection of triple-mRNA vaccinated individuals is associated with robust neutralization of the previous Omicron variants BA.1, BA.2, BA.2.12.1, and BA.4/BA.5 itself.. Our findings are consistent with a recent report showing strong cross-neutralization of Omicron BA.1, BA.2, Beta and Delta in sera from individuals vaccinated with BNT162b2 or an adenovirus-based vaccine that encountered subsequent BA.4 breakthrough infection (*24*). In line with those observations in humans we also found high GMTs against BA.4/5 and Omicron sublineage cross-neutralizing activity in sera of mice that were boosted with the monovalent Omicron BA.4/5 adapted vaccine following primary immunization with BNT162b2, whereas an Omicron BA.1 boost induced lower cross-neutralization of BA.4/5, BA.2, and BA.2.12.1. The bivalent BNT162b2/BA.4/5 boost elicited broad Omicron sublineage cross-neutralization, with a similar pattern of potentiation of pre-D3 titers against individual Omicron sublineages slightly below in magnitude of those elicited by the BA.4/5 monovalent booster. The bivalent BNT162b2/BA.1 booster elicited higher neutralizing titers against Omicron BA.2 and its descendants including BA.4/5 than the monovalent BA.1 booster. However, neutralization breadth was mostly lower than that mediated by the mono- and the bivalent BA.4/5 booster. Together these findings suggest that exposure to Omicron BA.4/5 S glycoprotein confers robust neutralization activity against the currently circulating Omicron sublineage VOCs and may do so against potential future VOCs, should they be related to those.

In naïve mice we found that immunization with the BNT162b2/BA.4/5 bivalent vaccine elicits strong neutralizing antibodies against BA.4/5, previous Omicron sublineages as well as non-Omicron VOCs including the wild-type strain and is superior to the monovalent BA.4/5 in terms of both breadth and GMTs. Accordingly, for unvaccinated individuals who have not been previously infected with SARS-CoV-2, e.g., the young pediatric population, a bivalent vaccine approach may be particularly suitable.

There are several limitations to our study. Our investigation of Omicron-convalescents is a retrospective analysis of cohorts of small sample size. These cohorts are not balanced with regard to demographic characteristics such as age and sex of individuals and are not fully aligned in terms of intervals between vaccine doses, between the last vaccine dose and infection as well as between the most recent antigen exposure and blood sampling. While these factors differ only mildly across cohorts, they may confound magnitude and breadth of neutralizing activity. Therefore, we included GMT ratios to report on neutralization breadth independently of the magnitude of neutralizing antibody titers. The live SARS-CoV-2 neutralization assay may be compromised by variant-intrinsic differences in cell entry or replication. Therefore, we enlisted the pseudovirus neutralization assay, where the variant S glycoprotein used for pseudotyping, i.e. the primary target of neutralizing antibodies, is the only variable. While the mouse studies were conducted with a relatively small sample size and showed moderate variability in some analyses, the main results of the booster study in mice were again consistent across pseudovirus and live SARS-CoV-2 neutralization assay platforms. Further studies investigating long-lived plasma cell, memory B cell, and T cell immunity will provide further insights into the mechanisms underlying neutralization breadth of monovalent and bivalent Omicron BA.4/5 vaccines.

Our data suggest that a mono- or bivalent BA.4/BA.5 S glycoprotein adapted booster vaccine neutralizes the prevalent BA.4/BA.5 VOCs better than a vaccine based on a previously dominant Omicron variant such as BA.1. Given the global predominance and high transmissibility of Omicron BA.5 (*7*, *8*, *10*, *25*), it is possible that new descendants with further growth advantage emerge that may retain partial or full susceptibility to an Omicron BA.4/BA.5-adapted vaccine. A recent report showed partial susceptibility of descendant strains BA.4.6, BA.5.6, or BA.5.9 to neutralization by BA.4/BA.5 convalescent sera (*26*). However, as it is unpredictable how the virus will further evolve, a bivalent wild-type/Omicron BA.4/5-adapted booster may have enhanced neutralization breadth compared to the monovalent BA.4/5 vaccine, making it suitable to address potential new variants that are antigenically distinct from Omicron BA.4/5 but closer related to the wild-type strain. Monitoring of virus evolution and epidemiological landscapes remains instrumental to inform further vaccine adaptations in response to emerging threats.

## MATERIALS AND METHODS

### Human study design, recruitment of participants and sample collection

The objective of this study was to investigate the effect of Omicron BA.4/BA.5 breakthrough infection on the cross-variant neutralization capacity of human sera. We assessed neutralizing activity in immune sera from triple-mRNA (BNT162b2/mRNA-1273)-vaccinated individuals with a confirmed subsequent SARS-CoV-2 breakthrough infection, which either occurred in a period of Omicron BA.4/BA.5 lineage-dominance in Germany (mid-June to mid-July 2022) or was variant-confirmed (BA.4 or BA.5) by genome sequencing (mRNA-Vax^3^ + BA.4/5) (fig. S2 and Tables S1 and S2). Three cohorts were included for reference: Immune sera of triple mRNA vaccinated individuals with a confirmed subsequent SARS-CoV-2 breakthrough infection in a period of Omicron BA.2 lineage-dominance (March to May 2022; mRNA-Vax^3^ + BA.2), a period of Omicron BA.1 lineage-dominance (November 2021 to mid-January 2022; mRNA-Vax^3^ + BA.1), and triple-BNT162b2-vaccinated individuals that were SARS-CoV-2-naïve (nucleocapsid seronegative) at the time of sample collection (BNT162b2^3^). Serum neutralizing capability was characterized using pseudovirus and live SARS-CoV-2 neutralization assays. Data for the reference cohorts were previously published (*14*, *17*).

Convalescent individuals were recruited from University Hospital, Goethe University Frankfurt as part of a non-interventional study (protocol approved by the Ethics Board of the University Hospital [No. 2021–560]) researching patients that had experienced Omicron breakthrough infection following vaccination for COVID-19. Individuals from the BNT162b2^3^ cohort provided informed consent as part of their participation in the Phase 2 trial BNT162–17 (NCT05004181).

The infections of 5 BA.4/5 convalescent, 1 BA.2 convalescent, and 4 BA.1 convalescent participants in this study were confirmed by genome sequencing (Table S2 and (*14*, *17*)).

All participants had no documented history of SARS-CoV-2 infection prior to vaccination. Participants were free of symptoms at the time of blood collection.

Serum was isolated by centrifugation of drawn blood at 2000 x g for 10 minutes and cryopreserved until use.

### Mouse studies

All mouse studies were performed at BioNTech SE, and protocols were approved by the local authorities (local welfare committee) and conducted according to Federation of European Laboratory Animal Science Associations recommendations. Study execution and housing were in compliance with the German Animal Welfare Act and Directive 2010/63/EU. Mice were kept in individually ventilated cages with a 12-h light/dark cycle, controlled environmental conditions (22 ± 2°C, 45% to 65% relative humidity) and under specific-pathogen-free (SPF) conditions. Food and water were available ad libitum. Before initiation of the studies, animals were assessed for their general condition, for example for deviations from age-adequate weight, fur condition, grooming activity, posture and locomotion. Only mice with no disqualifying health conditions and SPF hygiene status were selected for testing procedures. Group sizes were determined as n = 8 (non-parametric test) or n = 5 (parametric test) to achieve statistical power of 80% (P = 0.8) and a significance level of 5% (α = 0.05).

For immunization, female BALB/c mice (Janvier) (9–21 weeks old) were randomly allocated to groups. BNT162b2 and Omicron-based vaccines candidates were diluted in 0.9% NaCl and 1 μg of the vaccine candidate was injected into the gastrocnemius muscle at a volume of 20 μl under isoflurane anesthesia. For the mouse booster study, mice were immunized twice (day 0 and 21) with BNT162b2. Third immunization with BNT162b2 and Omicron-based vaccine candidates occurred at day 104 after study start. Peripheral blood was collected from the facial vein without anesthesia for interim bleedings shortly before (same day) the third immunization, and 7 and 21 days after the third immunization. Final bleeding was performed under isoflurane anesthesia from the retro-orbital venous plexus at day 35 after the third immunization. For the naïve mouse study, animals were immunized at day 0 and 21 with BNT162b2 and Omicron-based vaccines candidates. 14 days after second immunization, blood was drawn. For serum generation, blood was centrifuged for 5 min at 16,000xg and the serum was immediately used for downstream assays or stored at −20°C until time of use.

### VSV-SARS-CoV-2 S variant pseudovirus generation

A recombinant replication-deficient vesicular stomatitis virus (VSV) vector that encodes green fluorescent protein (GFP) and luciferase instead of the VSV-glycoprotein (VSV-G) was pseudotyped with SARS-CoV-1 S glycoprotein (UniProt Ref: P59594) or with SARS-CoV-2 S glycoprotein derived from either the wild-type strain (Wuhan-Hu-1, NCBI Ref: 43740568), or the Alpha, Delta, Omicron BA.1, BA.2, BA.2.12.1, or the BA.4/5 variant according to published pseudotyping protocols (*28*). A diagram of SARS-CoV-2 S glycoprotein alterations is shown in fig. S9a and a separate alignment of S glycoprotein alterations in Omicron VOCs is displayed in fig. S1.

In brief, HEK293T/17 monolayers (ATCC CRL-11268) cultured in Dulbecco’s modified Eagle’s medium (DMEM) with GlutaMAX (Gibco) supplemented with 10% heat-inactivated fetal bovine serum (FBS [Sigma-Aldrich]) (referred to as medium) were transfected with Sanger sequencing-verified SARS-CoV-1 or variant-specific SARS-CoV-2 S expression plasmid with Lipofectamine LTX (Life Technologies, cat. no. 15338500) following the manufacturer’s instructions. At 24 hours after transfection, the cells were infected at a multiplicity of infection (MOI) of three with VSV-G complemented VSVΔG vector. After incubation for 2 hours at 37°C with 7.5% CO_2_, cells were washed twice with phosphate buffered saline (PBS) before medium supplemented with anti-VSV-G antibody (clone 8G5F11, Kerafast Inc., cat. no. EB0010) was added to neutralize residual VSV-G-complemented input virus. VSV-SARS-CoV-2-S pseudotype-containing medium was harvested 20 hours after inoculation, passed through a 0.2 μm filter (Nalgene) and stored at −80°C. The pseudovirus batches were titrated on Vero 76 cells (ATCC CRL-1587) cultured in medium. The relative luciferase units induced by a defined volume of a SARS-CoV-2 wild-type strain S glycoprotein pseudovirus reference batch previously described in Muik et al. (*21*) that corresponds to an infectious titer of 200 transducing units (TU) per mL, was used as a comparator. Input volumes for the SARS-CoV-2 variant pseudovirus batches were calculated to normalize the infectious titer based on the relative luciferase units relative to the reference.

### Pseudovirus neutralization assay

Vero 76 cells were seeded in 96-well white, flat-bottom plates (Thermo Scientific) at 40,000 cells/well in medium 4 hours prior to the assay and cultured at 37°C with 7.5% CO_2_. Human and mouse serum samples were 2-fold serially diluted in medium with dilutions ranging from 1:5 to 1:30,720 (human sera), from 1:40 to 1:102,400 for mouse booster study (mouse sera; starting dilution was 1:40 [pre-D3], 1:200 [d7D3]) as well as 1:100 [d21D3, d35D3]) and from 1:100 to 1:15,360 in the naïve setting (mouse sera; starting dilution was 1:120 [monovalent vaccinated groups] and 1:100 [bivalent vaccinated groups]). VSV-SARS-CoV-2-S/VSV-SARS-CoV-1-S particles were diluted in medium to obtain 200 TU in the assay. Serum dilutions were mixed 1:1 with pseudovirus (n = 2 technical replicates per serum per pseudovirus) for 30 minutes at room temperature before being added to Vero 76 cell monolayers and incubated at 37°C with 7.5% CO_2_ for 24 hours. Supernatants were removed and the cells were lysed with luciferase reagent (Promega). Luminescence was recorded on a CLARIOstar Plus microplate reader (BMG Labtech), and neutralization titers were calculated as the reciprocal of the highest serum dilution that still resulted in 50% reduction in luminescence. Results for all pseudovirus neutralization experiments were expressed as geometric mean titers (GMT) of duplicates. If no neutralization was observed, an arbitrary titer value of half of the limit of detection [LOD] was reported. Neutralization titers in human sera are shown in Tables S3 to S6.

### Live SARS-CoV-2 neutralization assay

SARS-CoV-2 virus neutralization titers were determined by a microneutralization assay based on cytopathic effect (CPE) at VisMederi S.r.l., Siena, Italy. In brief, human and mouse serum samples were serially diluted 1:2 (n = 2 technical replicates per serum per virus; starting at 1:10 for human samples and starting at 1:50 [post-boost, day 139] for murine samples) and incubated for 1 hour at 37°C with 100 TCID_50_ of the wild-type-like SARS-CoV-2 virus strain 2019-nCOV/ITALY-INMI1 (GenBank: MT066156), Omicron BA.1 strain hCoV-19/Belgium/rega-20174/2021, sequence-verified Omicron BA.2 or sequence-verified BA.4 strain. A diagram of S glycoprotein alterations is shown in Fig. S9b. The 2019-nCOV/ITALY-INMI1 strain S glycoprotein is identical in sequence to the wild-type SARS-CoV-2 S (Wuhan-Hu-1 isolate). Vero E6 (ATCC CRL-1586) cell monolayers were inoculated with the serum/virus mix in 96-well plates and incubated for 3 days (2019-nCOV/ITALY-INMI1 strain) or 4 days (Omicron BA.1, BA.2 and BA.4 variant strain) to allow infection by non-neutralized virus. The plates were observed under an inverted light microscope and the wells were scored as positive for SARS-CoV-2 infection (i.e., showing CPE) or negative for SARS-CoV-2 infection (i.e., cells were alive without CPE). The neutralization titer was determined as the reciprocal of the highest serum dilution that protected more than 50% of cells from CPE and reported as GMT of duplicates. If no neutralization was observed, an arbitrary titer value of 5 (half of the LOD) was reported. Neutralization titers in human sera are shown in Tables S7 to S10.

### Statistical analysis

The statistical method of aggregation used for the analysis of antibody titers is the geometric mean and for the ratio of SARS-CoV-2 VOC titer and wild-type strain titer the geometric mean and the corresponding 95% confidence interval. The use of the geometric mean accounts for the non-normal distribution of antibody titers, which span several orders of magnitude. The Friedman test with Dunn’s correction for multiple comparisons was used to conduct pairwise signed-rank tests of group geometric mean neutralizing antibody titers with a common control group. The Kruskal-Wallis test with Dunn’s correction for multiple comparisons was used to conduct unpaired signed-rank tests of group GMT ratios. All statistical analyses were performed using GraphPad Prism software version 9.

## Supplementary Materials

### Other Supplementary Material for this manuscript includes the following:

Data file S1

MDAR Reproducibility Checklist

## Notes

View/request a protocol for this paper from *Bio-protocol*.
