# Supplementary material for: Exposure to BA.4/5 S protein drives neutralization of Omicron BA.1, BA.2, BA.2.12.1, and BA.4/5 in vaccine-experienced humans and mice
Source: Sci Immunol. 2022 Nov 15:eade9888. doi: 10.1126/sciimmunol.ade9888 (PMC9765452; doi:10.1126/sciimmunol.ade9888)
Supplement: Supplementary file 1 — Methods Figs. S1 to S9 Tables S1 to S10 [file sciimmunol.ade9888_sm.pdf]

Science Immunology
AAAS

Supplementary Materials for

Exposure to BA.4/5 S protein drives neutralization of Omicron BA.1, BA.2, BA.2.12.1, and BA.4/5 in vaccine-experienced humans and mice

Alexander Muik *et al.*

Corresponding author: Ugur Sahin, ugur.sahin@biontech.de

Sci. Immunol. **7**, eade9888 (2022)
DOI: 10.1126/sciimmunol.ade9888

The PDF file includes:

Methods
Figs. S1 to S9
Tables S1 to S10

Other Supplementary Material for this manuscript includes the following:

Data file S1
MDAR Reproducibility Checklist

Supplementary Methods

In vitro **transcription and lipid-nanoparticle (LNP) formulation of the RNA**

The BNT162b2 vaccine was designed on a background of S sequences from SARS-CoV-2 isolate Wuhan-Hu-1 (GenBank: MN908947.3) with pre-fusion conformation-stabilizing K986P and V987P mutations. Omicron BA.1 and Omicron BA.4/5 vaccine candidates were designed based on BNT162b2 including sequence changes as shown in fig. S3. RNA production as well as formulation were performed as previously described for BNT162b2 (*27*). The vaccine candidates were stored at -70 to -80°C at a concentration of 0.5 mg/mL until time of use.

In vitro **expression of RNAs and vaccines**

HEK293T cells were transfected with 0.15 µg BNT162b2 or Omicron-adapted vaccines (lipid-nanoparticle-formulated), or with vaccine RNAs using RiboJuice™ mRNA Transfection Kit (Merck Millipore, cat. no. TR-1013) according to the manufacturer's instructions and incubated for 18 h. Transfected HEK293T cells were stained with Fixable Viability Dye (eBioscience) and incubated with mouse Fc-tagged recombinant human ACE2 (Sino Biological, cat. no. 10108-H92H). A secondary donkey anti-mouse antibody conjugated with AF647 was used for detection of surface expression. Cells were fixed (Fixation Buffer, BioLegend) prior to flow cytometry analysis using a FACSCelesta flow cytometer (BD Biosciences, BD FACSDiva software version 8.0.1) and FlowJo software version 10.6.2 (FlowJo, BD Biosciences).

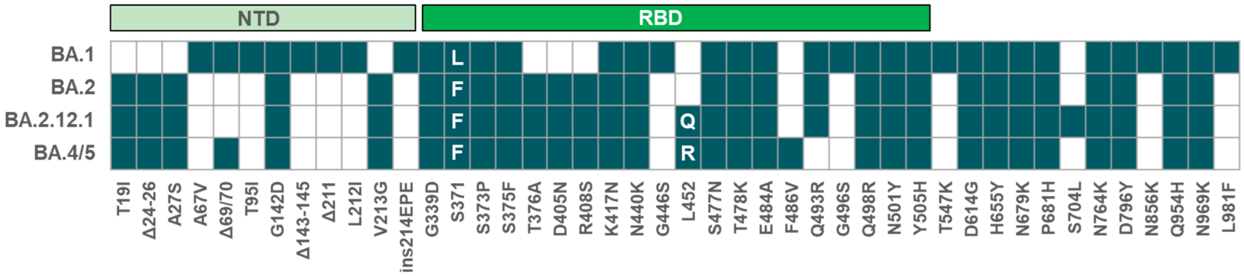

Fig. S1. Alterations of the spike glycoprotein amino acid sequence of SARS-CoV-2 Omicron sub-lineages.

White letters in boxes indicate the amino acid substitution per sub-lineage; Δ, deletion; ins, insertion; NTD, N-terminal domain; RBD, receptor-binding domain

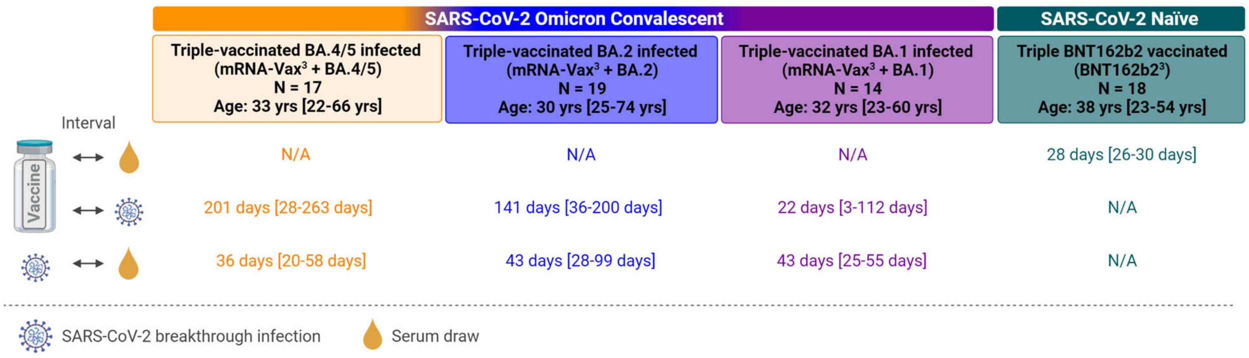

Fig. S2. Cohorts and sampling

Serum samples were drawn from four cohorts: individuals vaccinated with three doses of mRNA COVID-19 vaccine (BNT162b2/mRNA-1273 homologous or heterologous regimens) who subsequently had a breakthrough infection with Omicron BA.4/BA.5 (mRNA-Vax3 + BA.4/BA.5, orange). Three cohorts were included as reference: Triple-mRNA vaccinated individuals who experienced breakthrough infection with Omicron BA.2 (mRNA-Vax3 + BA.2, blue), or with BA.1 (mRNA-Vax3 + BA.1, purple), or individuals triple-vaccinated with BNT162b2 that were SARS-CoV-2-naïve at the time of sampling (BNT162b2^3, green). Breakthrough infections occurred at a time of respective VOC dominance (BA.4/BA.5: mid-June to mid-July 2022, BA.2: March to May 2022, BA.1: November 2021 to January 2021) and/or were variant confirmed by genome sequencing. For convalescent cohorts, relevant intervals between key events such as the most recent vaccination, SARS-CoV-2 infection, and serum isolation are indicated. All values specified as median-range. The age/gender composition of the cohorts is further detailed in Table S1. Participant-level information is provided for the mRNA-Vax^3 + BA.4/BA.5 in Table S2. Data for the reference cohorts mRNA-Vax^3 + BA.2, mRNA-Vax^3 + BA.1, and BNT162b2^3 were previously published (*14, 17*).

N/A, not applicable; Schematic was created with BioRender.com

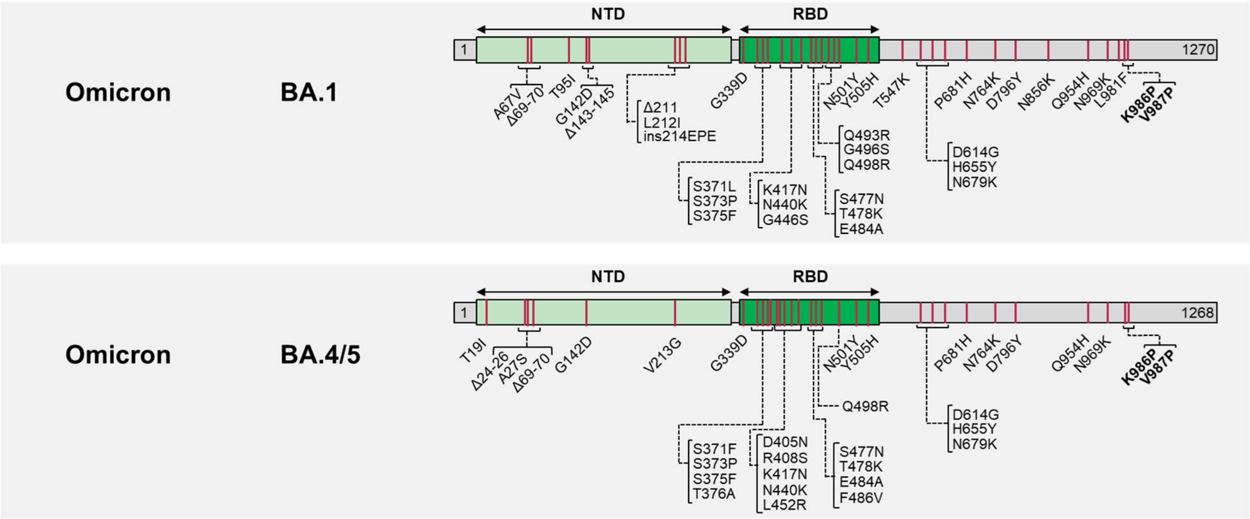

Fig. S3. Design of Omicron-adapted vaccines

The sequence of the Wuhan-Hu-1 isolate SARS-CoV-2 S glycoprotein (GenBank: QHD43416.1) was used as reference. Amino acid positions, amino acid descriptions (one letter code) and type of alterations (substitutions, deletions, insertions) are indicated. Pre-fusion stabilizing mutations are highlighted in bold. NTD, N-terminal domain; RBD, Receptor-binding domain, Δ, deletion; ins, insertion

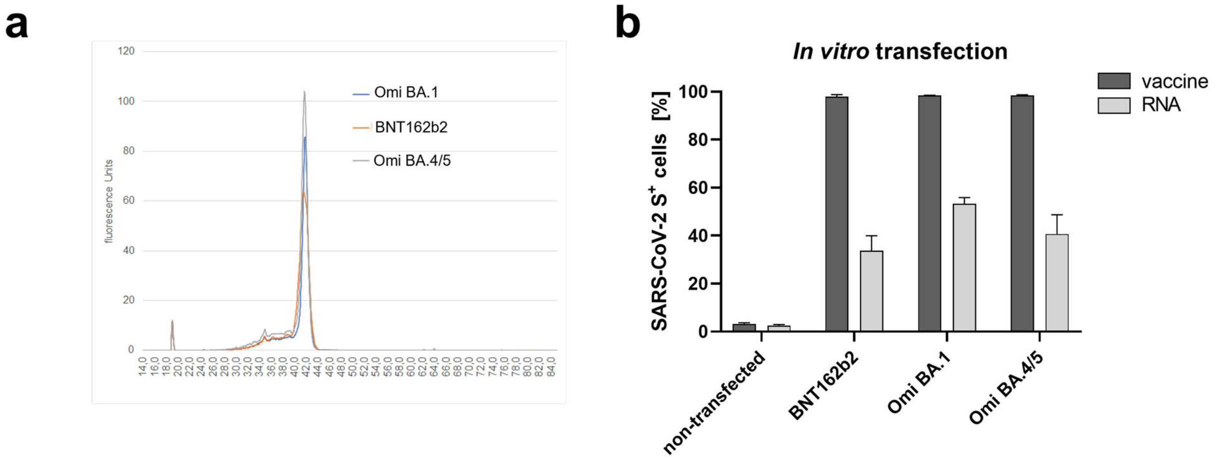

Fig. S4. Comparably high RNA purity and integrity, and *in vitro* expression of antigens encoded by Omicron-adapted vaccines

(a) Liquid capillary electropherograms of *in vitro* transcribed RNA samples merged into one graph. **(b)** Surface expression of SARS-CoV-2 Spike (S) glycoprotein on HEK293T cells upon transfection. HEK293T cells were transfected with S glycoprotein encoding mRNAs formulated as lipid nanoparticles (vaccine) or mixed with a commercial transfection reagent (RNA), or no vaccine/RNA (non-transfected). Surface expression was analyzed by flow cytometry using mFc-tagged human ACE2 as a detection reagent. Heights of bars indicate the means of n= 3 technical replicates.

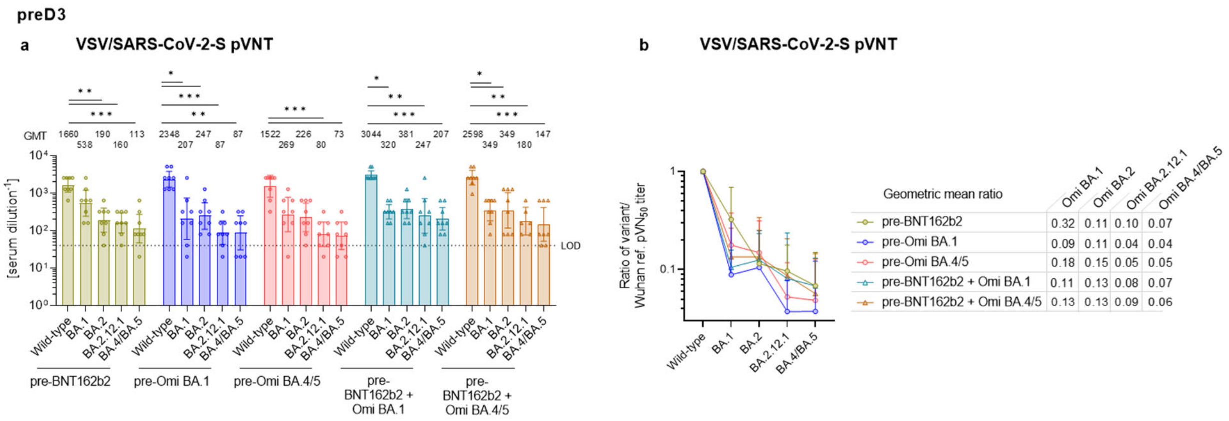

Fig. S5. Baseline magnitude and breadth of neutralizing activity against SARS-CoV-2 variants are comparable in BNT162b2-vaccinated mice prior to booster vaccination

BALB/c mice were injected intramuscularly with two doses of 1 µg BNT162b2 at a 21-day interval. Mice were allocated to groups (n=8) in preparation of administration of the booster dose of the indicated vaccines to be tested. Serum was collected from the mice on day 104 after the first vaccination, before booster vaccines were injected. **(a)** 50% pseudovirus neutralization (pVN_{50}) geometric mean titers (GMTs) against the indicated SARS-CoV-2 variants of concern (VOCs). Values above bars represent group GMTs. Error bars represent 95% confidence intervals. The non-parametric Friedman test with Dunn's multiple comparisons correction was used to compare the wild-type strain neutralizing group GMTs with titers against the indicated variants. Multiplicity-adjusted p values are shown. ***, P<0.01; **, P<0.01; *, P<0.05. **(b)** SARS-CoV-2 VOC pVN_{50} GMTs normalized against the wild-type strain pVN_{50} GMT (ratio VOC to wild-type). Group geometric mean ratios with 95% confidence intervals are shown. The non-parametric Kruskal-Wallis test with Dunn's multiple comparisons correction was used to compare the VOC GMT ratios between cohorts. Significance was not reached for any comparison. Serum was tested in duplicate. For titer values below the limit of detection (LOD),

LOD/2 values were plotted. BA.2.12.1 neutralization data from two mice in the pre-BNT162b2 + Omi BA.4/5 group were excluded due to inconsistent RLU measurements.

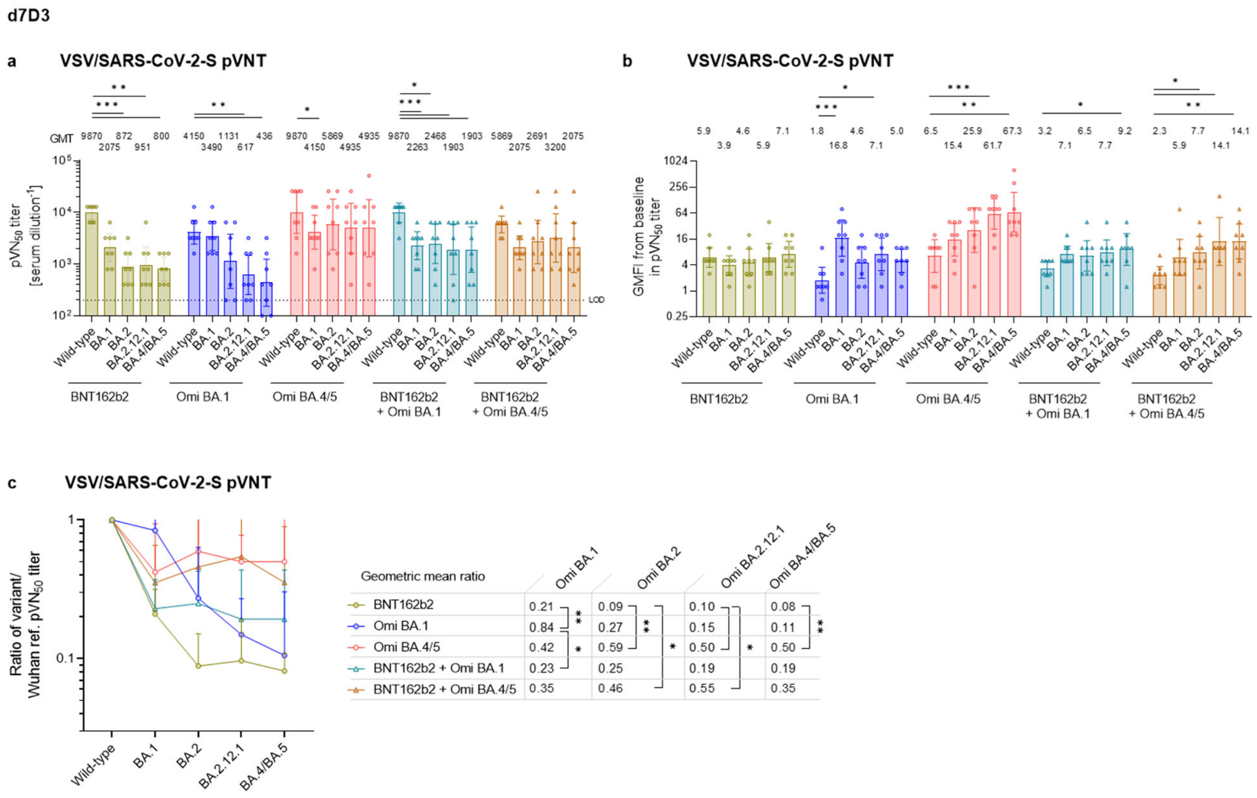

Fig. S6. Booster immunization with an Omicron BA.4/BA.5 S glycoprotein adapted mRNA vaccine mediates broad Omicron neutralization in mice 7 days after the booster

BALB/c mice (n=8) were injected intramuscularly with two doses of 1 µg BNT162b2 at a 21-day interval, and a third dose of either BNT162b2 (1 µg) or the indicated monovalent (1 µg) or bivalent (0.5 µg of each component) Omicron BA.1 or BA.4/5-adapted vaccines 104 days after the first vaccination. **(a)** 50% pseudovirus neutralization (pVN_{50}) geometric mean titers (GMTs) against the indicated SARS-CoV-2 variants of concern (VOCs) in sera collected 7 days after the third vaccination (d7D3). Values above bars represent group GMTs. **(b)** Geometric mean fold-increase (GMFI) of pVN_{50} titers on d7D3 relative to baseline titers before the third vaccination. Values above bars represent group GMFIs. The non-parametric Friedman test with Dunn's multiple comparisons correction was used to compare the wild-type strain neutralizing group

GMTs and group GMFI with those against the indicated variants. Multiplicity-adjusted p values are shown. ***, P<0.01; **, P<0.01; *, P<0.05. **(c)** SARS-CoV-2 VOC pVN_{50} GMTs normalized against the wild-type strain pVN_{50} GMT (ratio VOC to wild-type). Group geometric mean ratios are shown. The non-parametric Kruskal-Wallis test with Dunn's multiple comparisons correction was used to compare the VOC GMT ratios between cohorts. Significance levels are summarized as above. Serum was tested in duplicate. For titer values below the limit of detection (LOD), LOD/2 values were plotted. Error bars represent 95% confidence intervals.

d21D3

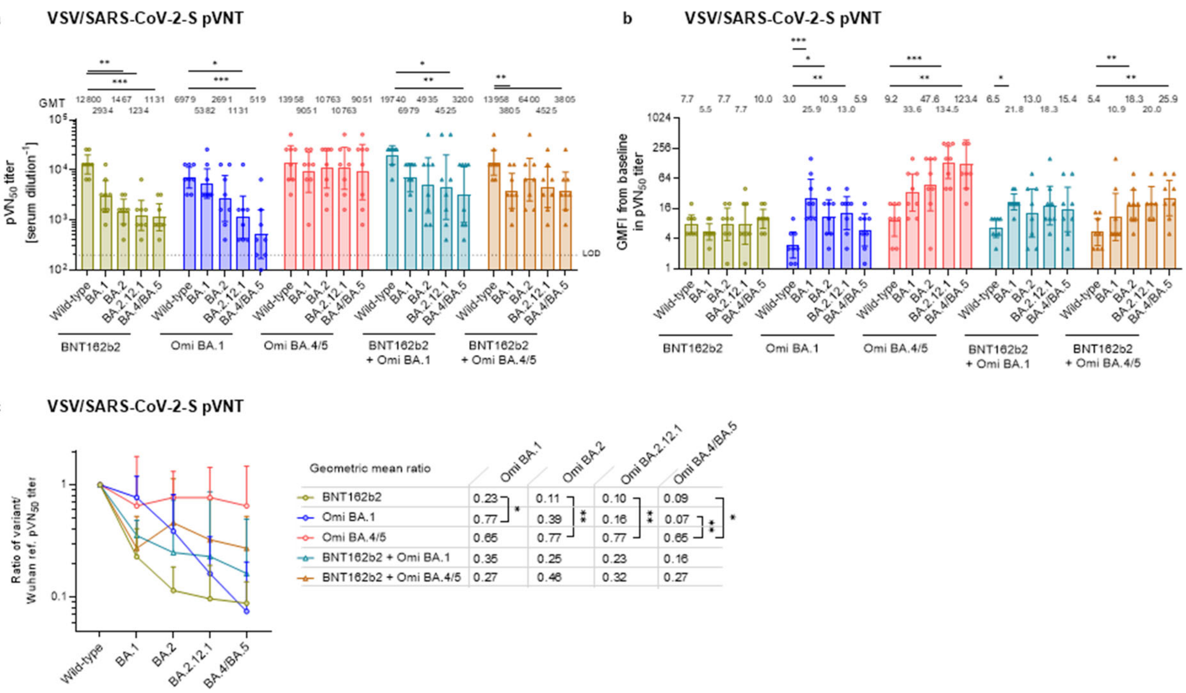

Fig. S7. Booster immunization with an Omicron BA.4/BA.5 S glycoprotein adapted vaccine mediates broad Omicron neutralization in mice 21 days after the booster

BALB/c mice (n=8) were injected intramuscularly with two doses of 1 µg BNT162b2 at a 21-day interval, and a third dose of either BNT162b2 (1 µg) or the indicated monovalent (1 µg) or bivalent (0.5 µg of each component) Omicron BA.1 or BA.4/5-adapted vaccines 104 days after the first vaccination. **(a)** 50% pseudovirus neutralization (pVN_{50}) geometric mean titers (GMTs) against the indicated SARS-CoV-2 variants of concern (VOCs) in sera collected 21 days after the third vaccination (d21D3). Values above bars represent group GMTs. **(b)** Geometric mean fold-increase (GMFI) of pVN_{50} titers on d21D3 relative to baseline titers before the third vaccination. Values above bars represent group GMFIs. The non-parametric Friedman test with Dunn's multiple comparisons correction was used to compare the wild-type strain neutralizing group GMTs and group GMFI with those against the indicated variants. Multiplicity-adjusted p values

are shown. ****, P<0.0001; ***, P<0.01; **, P<0.01; *, P<0.05. **(c)** SARS-CoV-2 VOC pVN50 GMTs normalized against the wild-type strain pVN_{50} GMT (ratio VOC to wild-type). Group geometric mean ratios are shown. The non-parametric Kruskal-Wallis test with Dunn's multiple comparisons correction was used to compare the VOC GMT ratios between cohorts. Significance levels are summarized as above. Serum was tested in duplicate. For titer values below the limit of detection (LOD), LOD/2 values were plotted. Error bars represent 95% confidence intervals.

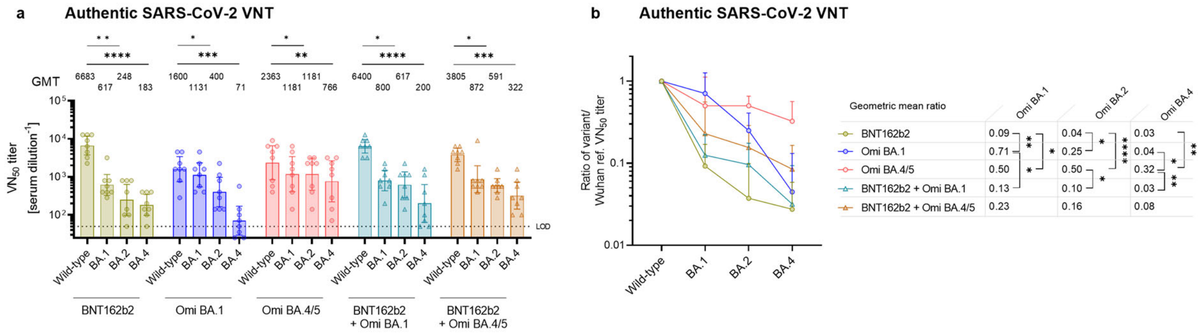

Fig. S8. Booster immunization with an Omicron BA.4/BA.5 S glycoprotein adapted mRNA-vaccine mediates broad Omicron neutralization 35 days after the booster

BALB/c mice (n=8) were injected intramuscularly with two doses of 1 µg BNT162b2 at a 21-day interval, and a third dose of either BNT162b2 (1 µg) or the indicated monovalent (1 µg) or bivalent (0.5 µg of each component) Omicron BA.1 or BA.4/5-adapted vaccines 104 days after the first vaccination. **(a)** 50% virus neutralization (VN_{50}) geometric mean titers (GMTs) against the indicated SARS-CoV-2 variants of concern (VOCs) in sera collected 35 days after the third vaccination (d35D3). Values above bars represent group GMTs. The non-parametric Friedman test with Dunn's multiple comparisons correction was used to compare the wild-type strain neutralizing group GMTs with those against the indicated variants. Multiplicity-adjusted p values are shown. ****, P<0.0001; ***, P<0.01; **, P<0.01; *, P<0.05. **(b)** SARS-CoV-2 VOC VN_{50} GMTs normalized against the wild-type strain VN_{50} GMT (ratio VOC to wild-type). Group geometric mean ratios are shown. The non-parametric Kruskal-Wallis test with Dunn's multiple comparisons correction was used to compare the VOC GMT ratios between cohorts. Significance levels are summarized as above. Serum was tested in duplicate. For titer values below the limit of detection (LOD), LOD/2 values were plotted. Error bars represent 95% confidence intervals.

a

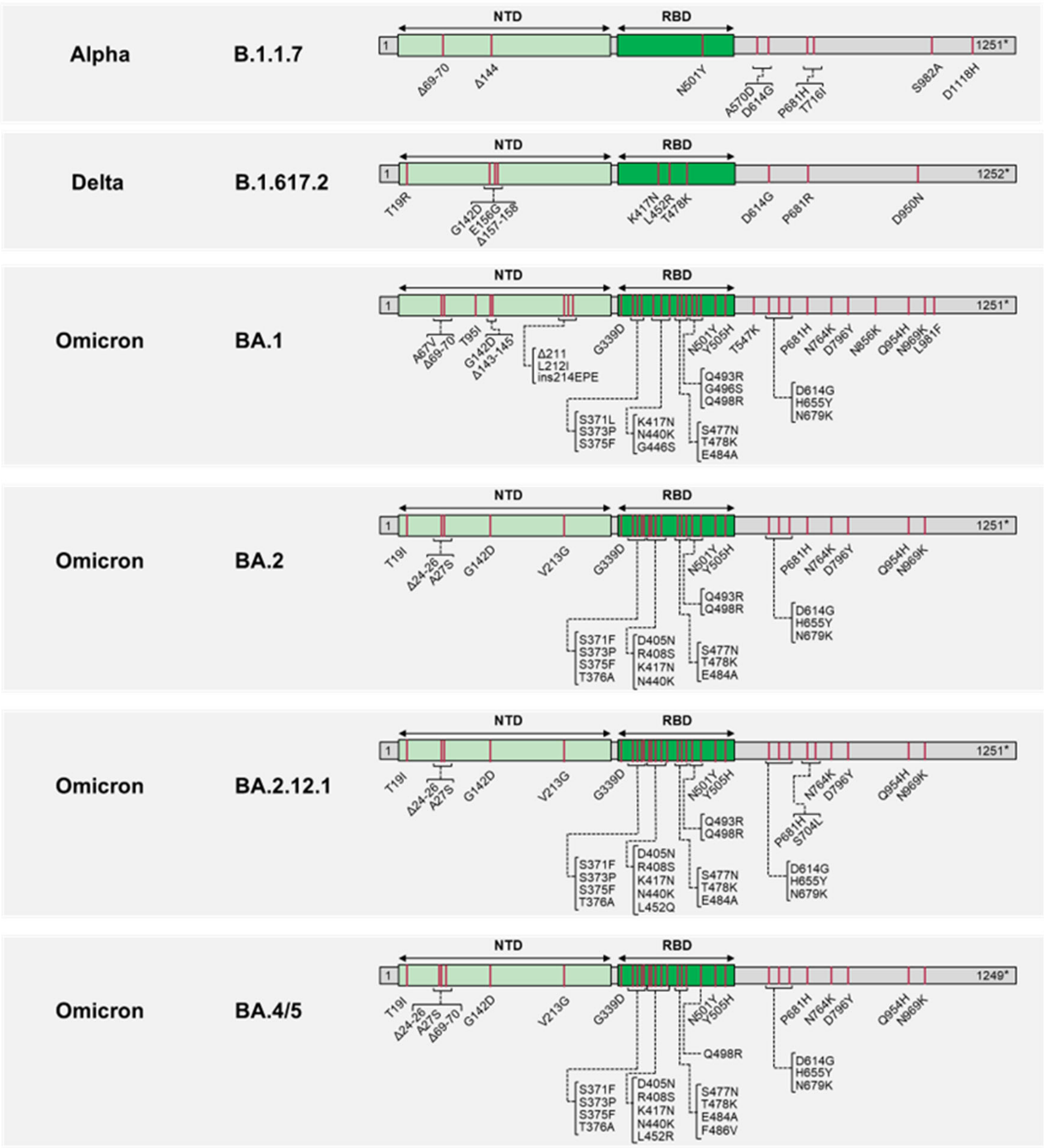
Alpha
B.1.1.7
Delta
B.1.617.2
Omicron
BA.1
Omicron
BA.2
Omicron
BA.2.12.1
Omicron
BA.4/5
NTD
RBD

b

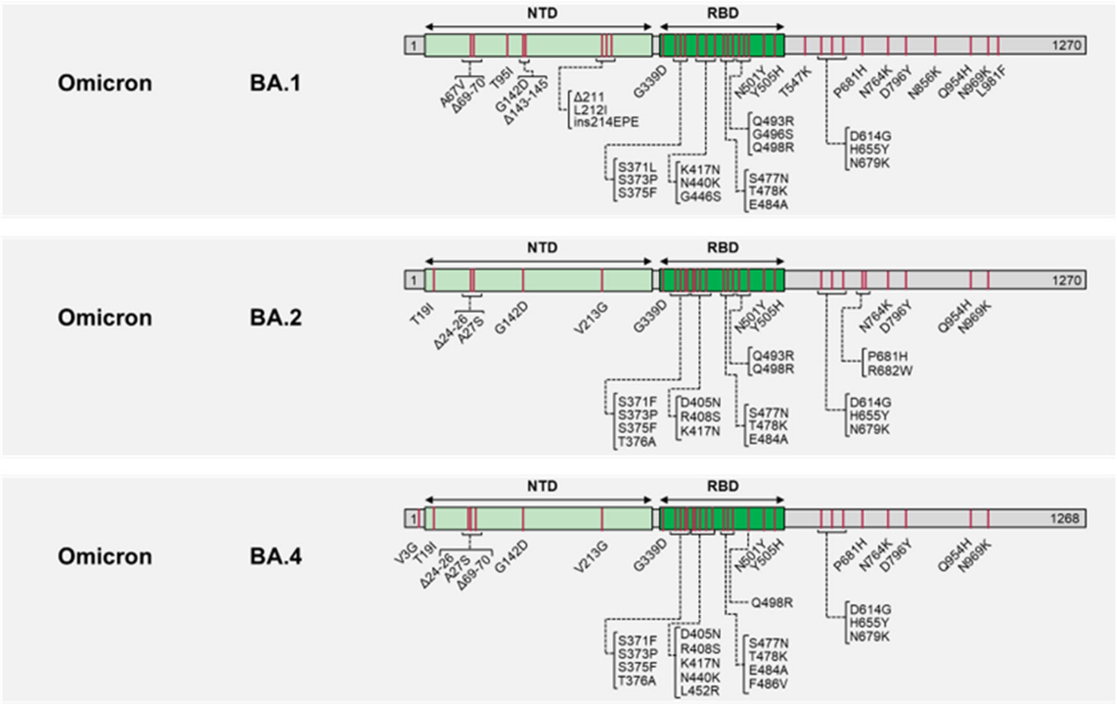

Fig. S9. Characterization of SARS-CoV-2 S glycoproteins used in the assays based on (a) VSV-SARS-CoV-2 variant pseudoviruses and (b) live authentic SARS-CoV-2.

The sequence of the Wuhan-Hu-1 isolate SARS-CoV-2 S glycoprotein (GenBank: QHD43416.1) was used as reference. Amino acid positions, amino acid descriptions (one letter code) and type of alterations (substitutions, deletions, insertions) are indicated. NTD, N-terminal domain; RBD, Receptor-binding domain, Δ, deletion; ins, insertion; *, Cytoplasmic domain truncated for the C-terminal 19 amino acids.

Table S1. Vaccinated individuals analyzed for neutralizing antibody responses.

Characteristic	mRNA-Vax[3] + BA.4/BA.5 (n=17)	mRNA-Vax[3] + BA.2 (n=19)	mRNA-Vax[3] + BA.1 (n=14)	BNT162b2[3] (n=18)
Sex, n (%)				
Male	7 (41)	7 (37)	11 (79)	9 (50)
Female	10 (59)	12 (63)	3 (21)	9 (50)
Age, median (range)	33 (22-66)	30 (25-74)	32 (23-60)	38 (23-54)
Age group at vaccination, n (%)				
18-55 yrs	15 (88)	15 (79)	12 (86)	18 (100)
56-85 yrs	2 (12)	4 (21)	2 (14)	0 (0)
SARS-CoV-2 status, n (%)				
Positive	17 (100)*	19 (100)**	14 (100)[#]	0 (0)
Negative	0 (0)	0 (0)	0 (0)	18 (100)†
Unknown	0 (0)	0 (0)	0 (0)	0 (0)
Interval, median (range)				
Days between D1/D2	35 (21-45)	42 (15-43)	38 (20-92)	‡
Days between D2/D3	193 (156-305)	184 (152-259)	192 (154-256)	202 (181-266)
Days until serum draw after D3	N/A	N/A	N/A	28 (26-30)
Days between last dose/infection	201 (28-263)	141 (36-200)	25 (3-112)	N/A
Days until serum draw after infection	36 (20-58)	43 (28-99)	43 (25-55)	N/A

N/A, not applicable; D, dose; yrs, years; n, number.
*, 5 cases were sequence verified BA.5 infections, 2 cases were family members of individuals with sequence-verified BA.5 infections, 8 individuals experienced SARS-CoV-2 breakthrough infection between June-July 2022, at which time the BA.4 or BA.5 lineage was dominant in Germany
**, Individuals experienced SARS-CoV-2 breakthrough infections between March and May 2022, during which period the BA.2 lineage was dominant in Germany. In two cases, BA.2 variant infection was confirmed by sequencing.
[#], Omicron infection PCR-confirmed at time of recruitment to the research study. Individuals experienced SARS-CoV-2 breakthrough infections between November 2021 and January 2022, during which period the BA.1 lineage was dominant in Germany
†, No evidence of prior SARS-CoV-2 infection (based on COVID-19 symptoms/signs and SARS-CoV-2 PCR test)
‡, Participants received the primary 2-dose series of BNT162b2 vaccine as part of a governmental vaccination program and the interval between doses was not recorded

Table S2. Individuals triple vaccinated with mRNA COVID-19 vaccine and subsequently infected with Omicron BA.4/BA.5 (mRNA-Vax^3 + BA.4/BA.5).

Participant ID	Age	Sex	Vaccination	Date positive test	Omicron subtype	Dose 1-2 interval (days)	Dose 2-3 interval (days)	Positive test after last vaccination (days)	Blood draw after positive test (days)	Severity (WHO grade)
1	32	f	BNT^2/MOD	MAY2022	N/A#	45	157	203	35	1-2
2	30	f	BNT^3	JUN2022	BA.5*	42	163	159	36	1-2
3	29	m	BNT^3	MAY2022	BA.5*	42	157	194	30	1-2
4	66	m	BNT^2/MOD	JUN2022	N/A#	42	183	178	41	1-2
5	57	m	BNT^3	JUL2022	BA.5*	24	283	246	20	1-2
6	36	m	BNT^2/MOD	JUN2022	N/A	35	198	201	27	1-2
7	22	f	BNT^3	JUL2022	BA.5*	42	189	187	26	1-2
8	33	f	BNT^2/MOD	JUN2022	N/A	42	156	194	47	1-2
9	32	f	BNT^3	JUL2022	N/A	21	305	214	35	1-2
10	25	f	MOD^2/BNT	JUL2022	BA.5*	42	198	180	37	1-2
11	30	m	BNT^3	JUN2022	N/A	21	269	211	57	1-2
12	28	f	BNT^3	JUL2022	N/A	21	193	216	28	1-2
13	32	f	BNT/MOD^2	JUN2022	N/A	42	177	189	50	1-2
14	37	m	BNT^3	JUN2022	N/A	21	274	233	50	1-2
15	49	m	BNT^3	JUL2022	N/A	21	178	203	39	1-2
16	30	f	BNT^3	JUN2022	N/A	21	294	199	58	1-2
17	31	f	BNT^3	JUL2022	N/A	21	276	263	29	1-2
**Median**	**32**	**N/A**	**N/A**	**N/A**	**N/A**	**35**	**193**	**201**	**36**	**N/A**

*Sequence-verified Omicron variant. #Family member of individual infected with sequence-verified Omicron variant.
m, male; f, female; n/a, not available; N/A, not applicable
BNT, BioNTech/Pfizer BNT162b2; MOD, Moderna mRNA-1273; BNT^3, BNT162b2 three-dose series; MOD^2, mRNA-1273 two-dose series; MOD^3, mRNA-1273 three-dose series

Table S3. pVN_{50} values of sera collected from individuals with Omicron BA.4/BA.5 breakthrough infection (mRNA-Vax[3] + BA.4/BA.5)

Participant ID	pVN_{50}					
	Wild-type	Omicron BA.1	Omicron BA.2	Omicron BA.2.12.1	Omicron BA.4/5	SARS-CoV-1
1	480	120	240	240	480	40
2	1920	480	960	960	960	80
3	960	960	480	480	480	160
4	960	960	480	960	960	5
5	240	60	60	60	60	5
6	1920	960	960	960	480	320
7	960	240	480	480	240	10
8	960	240	240	240	240	5
9	3840	3840	3840	3840	1920	40
10	960	480	480	960	480	5
11	1920	1920	1920	1920	960	10
12	960	480	1920	480	960	10
13	960	960	960	960	960	20
14	960	480	480	960	240	10
15	3840	1920	3840	1920	3840	80
16	480	120	120	120	60	5
17	960	240	480	480	960	5

Table S4. pVN$_{50}$ values of sera collected from SARS-CoV-2-naïve triple-vaccinated individuals (BNT162b2^{3})

Participant ID	pVN$_{50}$					
	Wild-type	Omicron BA.1	Omicron BA.2	Omicron BA.2.12.1	Omicron BA.4/5	SARS-CoV-1
18	160	80	160	80	40	5
19	640	160	320	160	40	40
20	5120	1280	1280	640	640	40
21	320	160	160	80	40	20
22	640	320	160	40	40	20
23	320	160	160	80	40	10
24	320	160	160	80	80	10
25	320	160	160	160	80	20
26	160	40	80	40	40	20
27	320	160	60	160	80	20
28	1280	640	640	640	320	80
29	40	5	40	10	5	5
30	320	80	160	80	40	20
31	160	80	160	80	40	20
32	320	320	320	160	160	20
33	640	160	320	80	80	40
34	2560	640	640	320	320	80
35	320	160	320	80	80	20

Table S5. pVN_{50} values of sera collected from individuals with Omicron BA.1 breakthrough infection (mRNA-Vax^3 + BA.1)

Participant ID	pVN_{50}					
	Wild-type	Omicron BA.1	Omicron BA.2	Omicron BA.2.12.1	Omicron BA.4/5	SARS-CoV-1
36	1920	1920	960	640	320	120
37	960	480	480	640	160	120
38	3840	1920	1920	1280	640	960
39	960	960	960	640	160	120
40	480	480	480	640	40	20
41	1920	960	1920	640	320	40
42	960	1920	480	320	80	80
43	960	480	480	320	80	5
44	480	480	480	320	80	60
45	1920	3840	1920	1280	2560	120
46	3840	3840	3840	2560	2560	120
47	7680	15360	3840	2560	1280	480
48	480	60	120	40	40	30
49	1920	960	960	640	640	60

Table S6. pVN_{50} values of sera collected from individuals with Omicron BA.2 breakthrough infection (mRNA-Vax[3] + BA.2)

Participant ID	pVN_{50}					
	Wild-type	Omicron BA.1	Omicron BA.2	Omicron BA.2.12.1	Omicron BA.4/5	SARS-CoV-1
50	480	240	480	160	120	5
51	3840	3840	3840	2560	960	40
52	480	240	240	240	120	10
53	240	120	240	120	120	5
54	1920	240	480	480	240	20
55	480	240	480	240	240	5
56	960	960	960	480	480	5
57	960	960	960	960	480	20
58	1920	960	1920	1920	480	20
59	960	240	480	240	120	10
60	960	240	480	480	480	10
61	1920	240	1920	1920	1920	5
62	960	960	960	480	1920	20
63	240	120	240	240	120	40
64	960	960	960	960	960	80
65	3840	1920	3840	960	480	640
66	960	240	480	480	240	20
67	960	960	960	960	960	160
68	3840	1920	1920	960	480	160

Table S7. VN_{50} values of sera collected from individuals with Omicron BA.4/BA.5 breakthrough infection (mRNA-Vax^3 + BA.4/BA.5)

Participant ID	VN_{50}			
	Wild-type	Omicron BA.1	Omicron BA.2	Omicron BA.4
1	160	113	160	113
2	905	320	640	453
3	226	160	320	453
4	905	320	453	453
5	113	20	40	40
6	640	226	2560	640
7	640	160	640	160
8	226	40	320	160
9	3620	1280	5120	1810
10	640	226	453	453
11	1280	640	1280	640
12	905	160	320	160
13	640	226	453	226
14	453	226	160	113
15	1810	1280	1280	1280
16	113	57	160	57
17	453	160	453	113

Table S8. VN_{50} values of sera collected from SARS-CoV-2-naïve triple-vaccinated individuals ($BNT162b2^3$)

Participant ID	VN_{50}			
	Wild-type	Omicron BA.1	Omicron BA.2	Omicron BA.4
18	226	40	40	28
19	640	160	80	28
20	3620	453	905	226
21	226	40	80	28
22	226	57	40	20
23	640	80	57	40
24	453	80	80	28
25	640	160	113	40
26	160	28	40	20
27	640	80	113	40
28	3620	453	453	160
29	28	5	10	5
30	453	40	80	20
31	226	57	57	28
32	905	160	113	40
33	1280	113	113	40
34	3620	1280	453	160
35	905	80	113	40

Table S9. VN_{50} values of sera collected from individuals with Omicron BA.1 breakthrough infection (mRNA-Vax[3] + BA.1)

Participant ID	VN_{50}			
	Wild-type	Omicron BA.1	Omicron BA.2	Omicron BA.4
36	453	640	905	226
37	453	453	453	80
38	1810	1810	1280	453
39	453	453	640	160
40	453	640	320	57
41	640	640	905	160
42	320	453	640	160
43	905	320	453	80
44	320	640	453	80
45	1280	1280	2560	640
46	5120	1280	3620	640
47	5120	5120	3620	453
48	160	40	113	7
49	1280	905	905	320

Table S10. VN_{50} values of sera collected from individuals with Omicron BA.2 breakthrough infection (mRNA-Vax^3 + BA.2)

Participant ID	VN_{50}			
	Wild-type	Omicron BA.1	Omicron BA.2	Omicron BA.4
50	320	226	320	113
51	3620	3620	3620	905
52	320	160	226	113
53	160	80	226	40
54	905	226	640	226
55	160	226	160	113
56	640	640	640	226
57	640	905	453	226
58	905	640	1280	320
59	453	226	320	160
60	320	160	640	226
61	1280	320	1280	640
62	453	453	640	320
63	453	113	640	226
64	453	1280	640	226
65	2560	640	1810	640
66	226	226	453	113
67	640	226	453	226
68	1810	640	1810	320
